# Subcapsular renal transplant hematoma mimicking acute tubular necrosis

**DOI:** 10.1002/ccr3.7774

**Published:** 2023-08-06

**Authors:** Akel Rhea, Haddad Elias, Hachem Kamal

**Affiliations:** ^1^ Medical Imaging Department Hotel Dieu de France Hospital Beirut Lebanon

**Keywords:** acute tubular necrosis, Doppler, renal transplant, subscapular hematoma, ultrasound

## Abstract

Kidney transplantation is the treatment of choice for patients with end‐stage renal disease. However, it is associated with serious potential complications, one of which is the subcapsular renal transplant hematoma. Ultrasound is the major imaging tools in the evaluation of early graft complications. We discuss the case of a patient who underwent a living‐donor kidney transplantation, complicated of acute kidney injury documented on serial blood tests with an elevation of creatinine levels and oliguria. Ultrasonography showed the presence of a subcapsular renal hematoma, associated with the same spectral characterizations of an acute tubular necrosis with a high resistive index on Color Doppler Ultrasonography Study of renal arteries. The patient underwent an emergent surgical evacuation of the subcapsular renal hematoma. A repeat ultrasonography showed the complete resolution of the subcapsular renal hematoma with normal resistive index. During the following days, diuresis was back to normal and serial blood tests showed normal levels of creatinine. This case report highlights the importance of Ultrasonography in detecting subcapsular hematomas that could be a reversible cause of acute kidney injury and acute tubular necrosis in the setting of renal transplant.

## Graphical abstract

Resistive index at 1 in a patient who underwent renal transplant complicated by subcapsular hematoma.

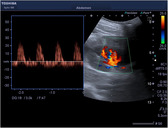

## 1 INTRODUCTION

Kidney transplantation is the treatment of choice for patients with end‐stage renal disease. However, it is associated with serious potential complications. In the immediate posttransplant period, ultrasound (US) is considered to be one of the major imaging tools in the evaluation of early graft complications. In fact, it is a noninvasive, nonionizing, effective, and reliable imaging modality that is accessible and portable and provides immediate results.

We discuss two cases of patients who underwent a living‐donor kidney transplantation, complicated by subcapsular hematoma mimicking acute tubular necrosis (ATN) on Color Doppler Ultrasonography Study (CDUS).

## 2 CASE PRESENTATION

Patient A is a 32‐year‐old woman, who was admitted to undergo an elective living‐donor kidney transplant. Medical history includes end‐stage renal disease due to focal segmental glomerulosclerosis (confirmed by histology) treated with conservative management, 8‐year history of chronic hypertension, and preeclampsia.

Family history includes a brother who underwent a living‐donor kidney transplant as a treatment for end‐stage renal disease secondary to membranous nephropathy.

Preoperative medical assessment was unremarkable. Vital signs were within normal limits, except for a blood pressure of 152/89 mmHg. Laboratory results showed a baseline serum creatinine levels of 877 μmol/L.

The surgery was performed without per‐operative complications, and the patient was transferred to the intensive care unit for observation.

Within 24 h of admission, the patient presented an acute oliguria, nonresponsive to fluid resuscitation. Physical examination showed a soft and nontender abdomen. Vital signs were normal. Serial laboratory results showed a progressive increase in serum creatinine levels, after reaching a nadir of 284 μmol/L. Acute renal rejection was suspected.

**FIGURE 1:**
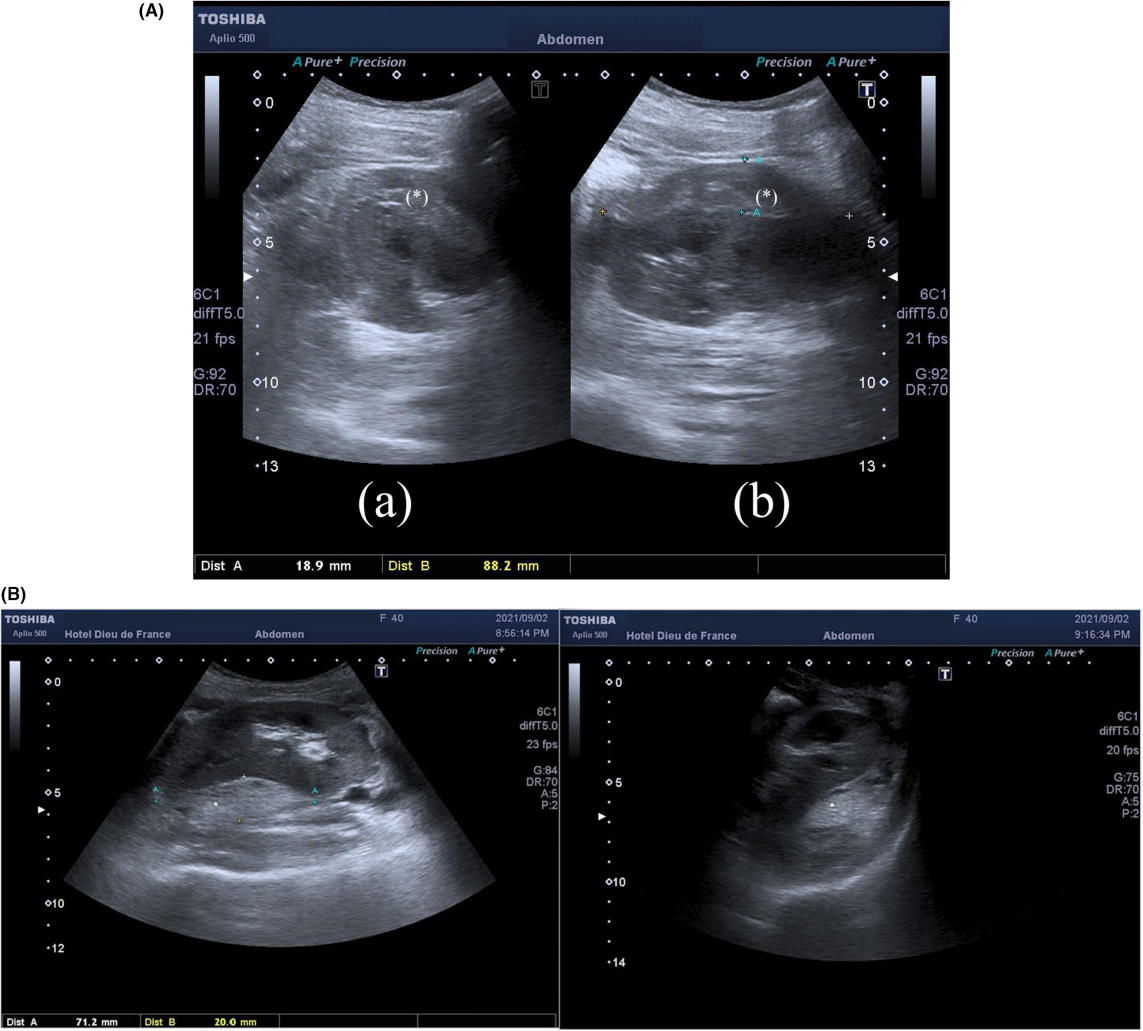
(A) Patient A: axial (a) and sagittal (b) views of the renal graft show a well‐differentiated kidney with an echogenic collection (*) encircling and deforming the parenchyma, compatible with a subcapsular hematoma. (B) Patient B: axial (a) and sagittal (b) views of the renal graft show a well‐differentiated kidney with an echogenic collection (*) encircling and deforming the parenchyma, compatible with a subcapsular hematoma.

**FIGURE 2:**
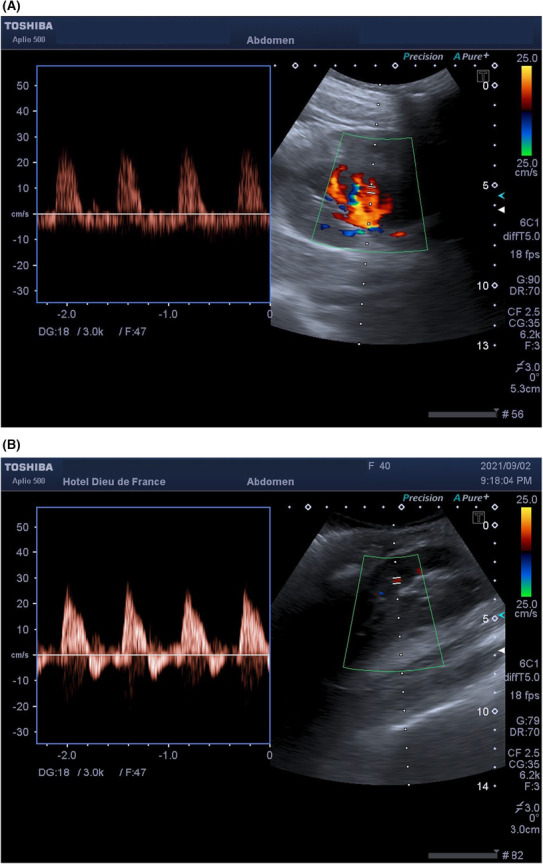
(A) Patient A: spectral waveform of intrarenal arteries shows a resistive flow with no diastolic component and diastolic reflux. The resistive index was evaluated at 1. (B) Patient B: spectral waveform of intrarenal arteries shows a resistive flow with no diastolic component and diastolic reflux. The resistive index was evaluated at 1.

Color‐Doppler sonography of transplant kidney performed urgently revealed a renal graft of normal size, measuring 10.8 cm, located in the right iliac fossa, with normal echostructure, and without hydronephrosis. Subcapsular renal hematoma of 22 mm thickness, extending on a length of 9 cm, with an estimated volume between 50 and 76 mL (Figure 1A), responsible for compression on the major part of the renal parenchyma, was noted. CDUS showed a high resistive flow, without a diastolic component, with a resistive index (RI) of 1 (Figure 2A). Diastolic reflux was noted. The graft vessels were patent, with no stenosis or thrombosis detected. Due to these findings, ATN was suspected.

**FIGURE 3:**
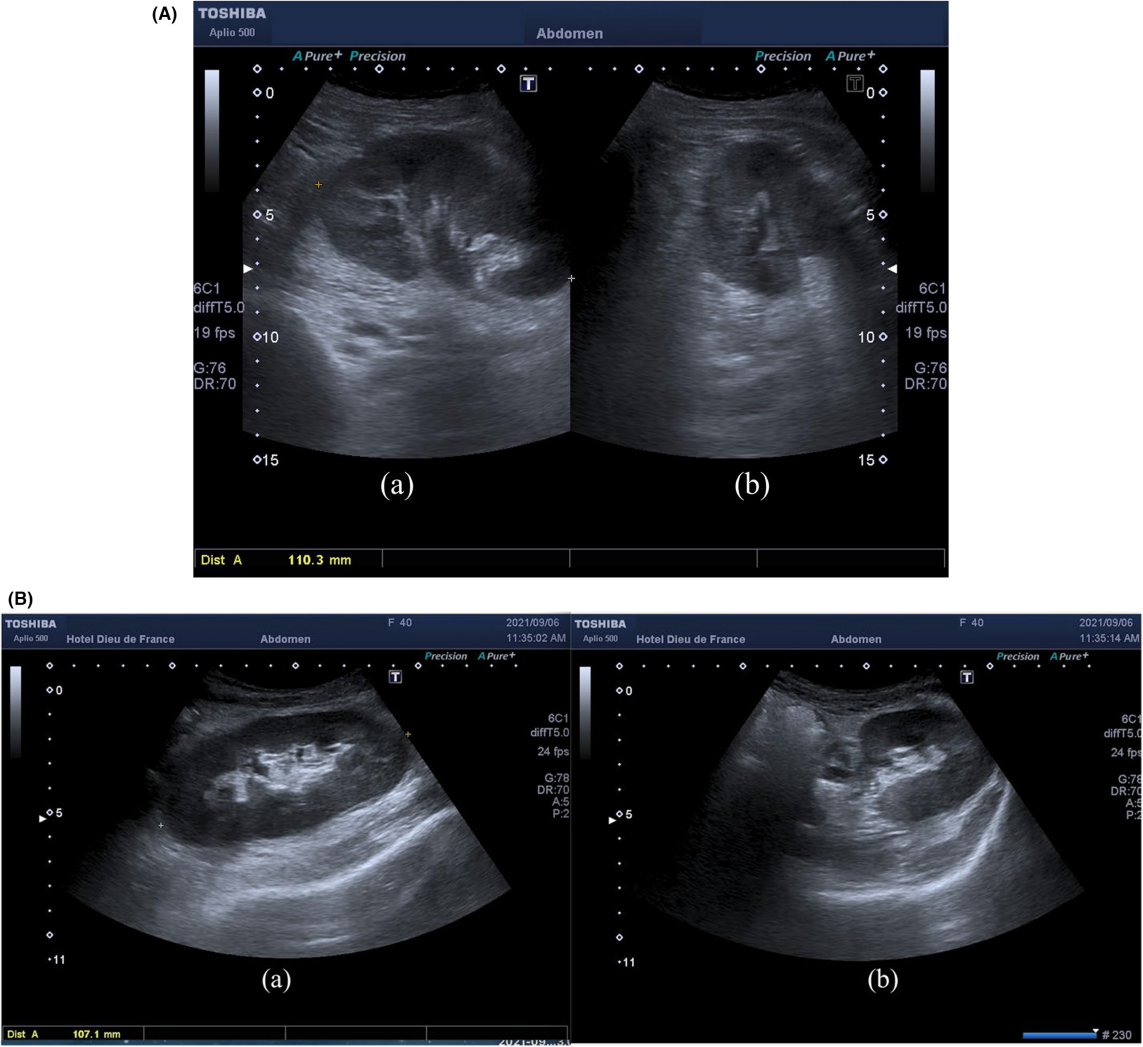
(A) Patient A: sagittal (a) and axial (b) views of the renal graft show a complete resolution of the subcapsular hematoma and its mass effect. (B) Patient B: sagittal (a) and axial (b) views of the renal graft show a complete resolution of the subcapsular hematoma and its mass effect.

**FIGURE 4:**
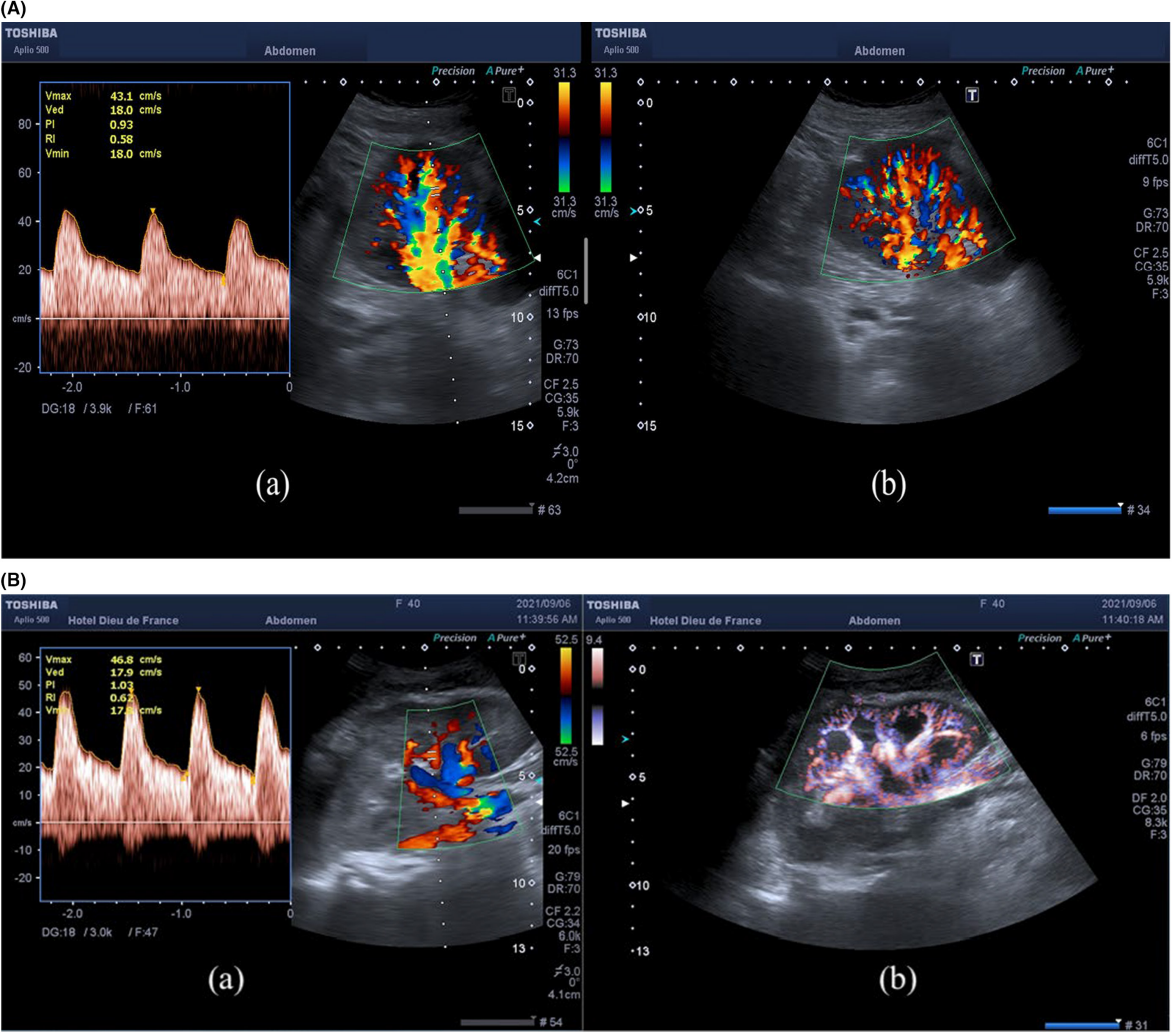
(A) Patient A: (a) spectral waveform of intrarenal arteries shows a complete return to normal of the resistive index, evaluated at 0.58. (b) Sagittal view of the graft shows a complete resolution of the mass effect and normal Color Doppler vascularization of the renal parenchyma. (B) Patient B: (a) spectral waveform of intrarenal arteries shows a complete return to normal of the resistive index, evaluated at 0.62. (b) Sagittal view of the graft shows a complete resolution of the mass effect and normal Color Energy vascularization of the renal parenchyma.

Following these results, the patient underwent an emergent surgical evacuation of the subcapsular renal hematoma. Subsequently and immediately, the diuresis was back to normal. A prompt repeat CDUS revealed complete resolution of the subcapsular hematoma (Figure 3A) with a normal RI, between 0.56 and 0.6 (Figure 4A). Serial laboratory results showed a continuous decline in serum creatinine levels until reaching normal levels of 80 μmol/L within 2 days only.

Thereafter, the patient had an uncomplicated in‐hospital stay and was discharged home 1 week later.

Patient B is a 40‐year‐old woman, who was admitted to undergo an elective living‐donor kidney transplant. Medical history includes end‐stage renal disease due to bilateral vesicoureteral reflux with recurrent urinary tract infections treated with bilateral ureteral reimplantation. Her family history was negative.

Preoperative medical assessment was unremarkable. Vital signs were within normal limits. Laboratory results showed a baseline serum creatinine levels of 687 μmol/L.

The surgery was performed without per‐operative complications, and the patient was transferred to the intensive care unit for observation.

Within 24 h of admission, the patient presented an acute oliguria, nonresponsive to fluid resuscitation. Physical examination showed a soft and nontender abdomen. Vital signs were normal. Serial laboratory results showed a progressive increase in serum creatinine levels, after reaching a nadir of 273 μmol/L. Acute renal rejection was suspected.

Color‐Doppler sonography of transplant kidney performed urgently revealed a renal graft of normal size, measuring 10.7 cm, located in the right iliac fossa, with normal echostructure, and without hydronephrosis. Subcapsular renal hematoma of 20 mm thickness, extending on a length of 7.1 cm (Figure 1B), responsible for compression on the major part of the renal parenchyma, was noted. CDUS showed a high resistive flow, without a diastolic component, with a resistive index (RI) of 1 (Figure 2B). Diastolic reflux was noted. The graft vessels were patent, with no stenosis or thrombosis detected. Due to these findings, ATN was suspected.

Following these results, the patient underwent an emergent surgical evacuation of the subcapsular renal hematoma. Subsequently and immediately, the diuresis was back to normal. A prompt repeat CDUS revealed complete resolution of the subcapsular hematoma (Figure 3B) with a normal RI, between 0.62 (Figure 4B). Serial laboratory results showed a continuous decline in serum creatinine levels until reaching normal levels of 64 μmol/L within 2 days only.

Thereafter, the patient had an uncomplicated in‐hospital stay and was discharged home 1 week later.

## 3 DISCUSSION

Kidney transplant is the treatment of choice for patients with end‐stage renal disease, however, it is classified as an intermediate–high‐risk surgery with serious potential complications. Examples of surgical and vascular complications include wound infection, hematoma, acute renal failure due to ATN, perirenal abscess, renal artery or vein thrombosis, urine leak, and many more.

Acute tubular necrosis is the most common cause of impaired renal function in the early posttransplantation period.^1^ It represents necrosis of tubular cells that commonly slough into the tubular lumen. The initial cause of ATN in transplant patients is usually related to the process of the transplant itself which causes ischemia to the kidney. In addition, reperfusion after the transplant may lead to oxygen‐free radical injury.^1^

The diagnosis of posttransplantation ATN is based on either inadequate and slow reduction of the serum creatinine level or oliguria through an early postoperative phase.^2^

Our patient suffered from acute oliguria during the early postoperative phase and rising serum creatinine levels, raising the suspicion of ATN.

A CDUS was performed on postoperative day 1 and revealed an RI of 1 with diastolic reflux, compatible with the diagnosis of ATN.

However, a subcapsular hematoma encircling the renal graft was concurrently seen, responsible for compression on the major part of the renal parenchyma, causing a mass effect and hyperpressure on the intrarenal vessels.

While the incidence of postoperative surgical‐site hemorrhage in kidney transplantation is relatively low, it may be associated with an increased risk of graft loss or death,^3^ thus, explaining the importance of early detection and adequate treatment.

The overall incidence of significant postoperative hematomas from renal transplants varies from 4% to 8%.^4^

The signs and symptoms of subcapsular hematoma in renal allografts vary depending on the duration and severity of the bleeding.^5^ The clinical presentation of patients with a single kidney and renal allografts includes flank pain/tenderness, decreased urine output, or acute renal failure,^5^ all of which were present in our two patients.

The subcapsular hematoma of our patient was totally evacuated in the operative room. Rapid clinical and biological improvements were noted. The diuresis was back to normal and serum creatinine levels reached normal values.

Only 2 days after complete evacuation of the hematoma, a repeat CDUS of the renal graft showed a normal RI, between 0.56 and 0.6, alongside complete resolution of the subcapsular hematoma.

Usually, ATN occurs right after the transplantation and resolves within 2 weeks but can last for up to 3 months. About 10%–30% of these patients require dialysis in the early stages.^1^

The rapid return to normal of the RI postoperatively, in addition to the rapid clinical and biological improvement within only 2 days of surgical evacuation of the hematoma, excludes the diagnosis of ATN. Thus, we can assume that the mass effect and the intrarenal hyperpressure caused by the subscapular renal hematoma were responsible for the abnormally elevated RI at 1, mimicking ATN.

Subcapsular renal hematomas can be managed conservatively. However, many case reports showed a conservative line of therapy to be life threatening.^5^ In fact, it could continue to increase with time and cause renal vein thrombosis. Surgical management should be in the first line of therapy when it comes to subcapsular renal hematomas with hemodynamic changes, in order to avoid any further complications and save the graft.

## 4 CONCLUSION

Despite the low incidence of postoperative renal hematoma following kidney transplantation, it can have serious complications affecting the outcome of the graft and even the patient. When the renal hematoma is subscapular with a mass effect on the renal parenchyma and intrarenal hyperpressure, CDUS can show a falsely elevated RI that can mimic ATN.

The mainstay of the treatment would be the emergent evacuation of the hematoma with postoperative imaging control.

## AUTHOR CONTRIBUTIONS

**Akel Rhea:** Data curation; investigation; writing – original draft. **Haddad Elias:** Formal analysis; supervision; writing – review and editing. **Hachem Kamal:** Conceptualization; methodology; project administration; supervision; validation; writing – review and editing.

## FUNDING INFORMATION

This study was financed with internal funds. No competing financial interests exist.

## CONFLICT OF INTEREST STATEMENT

The authors declare that they have no conflict of interest.

## CONSENT

Written informed consent was obtained from the patient to publish this report in accordance with the journal's patient consent policy.

## Data Availability

The data that support the findings of this study are available from the corresponding author upon reasonable request.
